# Supplementary material for: A yeast phenomic model for the influence of Warburg metabolism on genetic buffering of doxorubicin
Source: Cancer Metab. 2019 Oct 23;7:9. doi: 10.1186/s40170-019-0201-3 (PMC6806529; doi:10.1186/s40170-019-0201-3)
Supplement: Supplementary file 9 — Additional file 9. GO term-specific heatmaps for REMc/GTF-enriched clusters. GO term-specific heatmaps for significant GO process terms were generated as described in methods and Figs. 3 and 4. Any related child terms are presented in subsequent pages of the parent file name. GO terms with more than 100 children, with 2 or fewer genes annotated to the term, or a file size over 300KB are not shown. All heatmaps are generated with the same layout (see Figs. 3 and 4). [file 40170_2019_201_MOESM9_ESM.bz2 › Additional_File9_GOTermHeatmaps/Additional_File9_GOTermHeatmap/protein_targeting_to_mitochondrion.pdf]

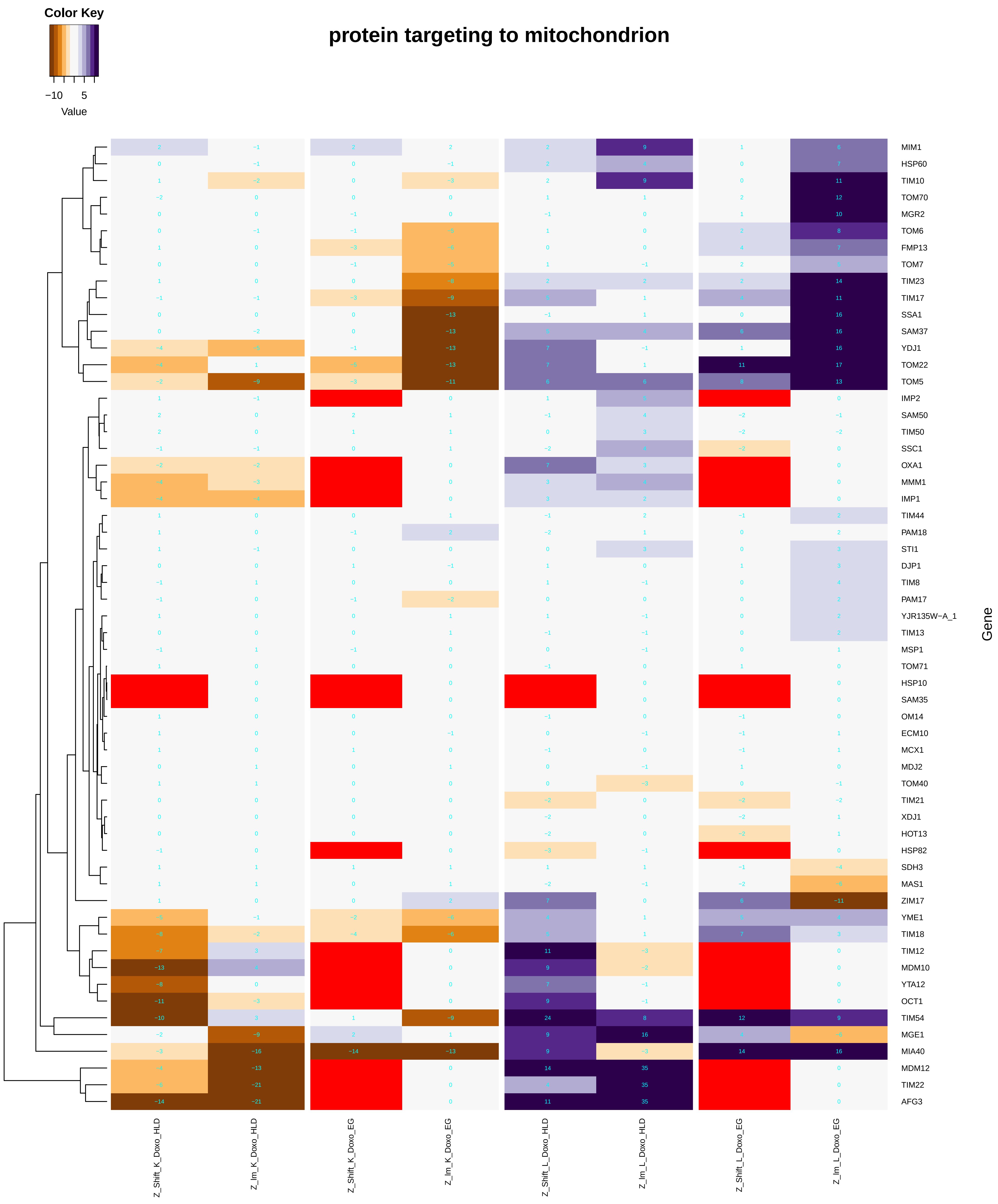

protein targeting to mitochondrion
Color Key
−10 5
Value
MIM1
HSP60
TIM10
TOM70
MGR2
TOM6
FMP13
TOM7
TIM23
TIM17
SSA1
SAM37
YDJ1
TOM22
TOM5
IMP2
SAM50
TIM50
SSC1
OXA1
MMM1
IMP1
TIM44
PAM18
STI1
DJP1
TIM8
PAM17
YJR135W−A_1
TIM13
MSP1
TOM71
HSP10
SAM35
OM14
ECM10
MCX1
MDJ2
TOM40
TIM21
XDJ1
HOT13
HSP82
SDH3
MAS1
ZIM17
YME1
TIM18
TIM12
MDM10
YTA12
OCT1
TIM54
MGE1
MIA40
MDM12
TIM22
AFG3
Gene
Z_Shift_K_Doxo_HLD
Z_lm_K_Doxo_HLD
Z_Shift_K_Doxo_EG
Z_lm_K_Doxo_EG
Z_Shift_L_Doxo_HLD
Z_lm_L_Doxo_HLD
Z_Shift_L_Doxo_EG
Z_lm_L_Doxo_EG
